# Immune biomarkers in circulating cells of NSCLC patients can effectively evaluate the efficacy of chemotherapy combined with anti-PD-1 therapy

**DOI:** 10.3389/fimmu.2025.1521708

**Published:** 2025-04-16

**Authors:** Jing Cao, Yuehua Zhang, Shenghu Guo, Zheng Wu, Xiaojin Guo, Rongze Zhang, Lei Zhang, Ya Liu, Xing Li, Chunwang Yang, Dongwei He, Lu Bai, Tingting Lv, Yong Xie, Chengjing Huang, Shuang Xiao, Anyi Deng, Jiawei Li, Jiaxing Zhu, Zhenghu Jia, Zhinan Yin, Zhiyu Wang

**Affiliations:** ^1^ Department of Immuno-Oncology, The Fourth Hospital of Hebei Medical University, Shijiazhuang, Hebei, China; ^2^ Department of Obstetrics and Gynecology, The First People’s Hospital of Foshan, Foshan, Guangdong, China; ^3^ Research and Development Department, Jiangxi Purui Biotechnology Co., Ltd., Ganzhou, Jiangxi, China; ^4^ The Biomedical Translational Research Institute, Faculty of Medical Science, Jinan University, Guangzhou, Guangdong, China; ^5^ Department of Oncology, The First Affiliated Hospital of Xinxiang Medical University, Weihui, Henan, China

**Keywords:** immune biomarkers, NSCLC, anti-PD-1 therapy, TIM-3, cytokines

## Abstract

**Introduction:**

The application of programmed cell death protein 1 (PD-1) antibodies has brought significant benefits to patients with non-small cell lung cancer (NSCLC). However, not all patients respond to PD-1 immune therapy. The aim of this study was to identify response biomarkers to predict the efficacy of chemotherapy combined with anti-PD-1 therapy in NSCLC patients.

**Methods:**

Thirty-two NSCLC patients receiving chemotherapy combined with anti-PD-1 therapy were recruited, and peripheral blood samples were collected before and after treatment. Flow cytometry was used to detect the proportions of circulating T-cell subsets, and cytokines in the blood serum were detected via ELISA.

**Results:**

The results revealed that, among the CR/PR group (CR, complete response; PR, partial response; n = 22), the proportions of CD3+TIM-3+PD-1+, CD3+CD4+TIM-3+PD-1+, and CD3+CD8+TIM-3+PD-1+, CD3+γδT+PD-1+, CD3+γδT+Vδ1+PD-1+, and CD3+γδT+Vδ2+PD-1+T cells were lower after treatment, with no significant differences found between the stable disease (SD) and progressive disease (PD) groups (n = 10). Some proinflammatory cytokines are highly expressed in patients with NSCLC.

**Discussion:**

This study suggests that monitoring changes in immune biomarkers in the circulating cells of NSCLC patients may help differentiate CR/PR patients from SD/PD patients, providing a potential new approach for assessing the efficacy of chemotherapy combined with anti-PD-1 therapy.

## Introduction

Both the incidence and mortality rates of lung cancer in China are the highest among those of malignant tumors. According to the latest statistics from the National Cancer Center, there are approximately 828,000 new cases each year, accounting for 24.6% of the total number of new cancer cases, with approximately 657,000 deaths annually, accounting for 29.71% of all cancer deaths (1). Non-small cell lung cancer (NSCLC) is the most common pathological type of lung cancer, accounting for approximately 85% of all lung cancer cases (2). Common treatment modalities for advanced NSCLC patients include maintenance therapy, chemotherapy, targeted therapy, and immunotherapy. Among all NSCLC patients, regardless of treatment group, the objective response rate is approximately 20%, with a median response duration of approximately 12 months (3–5). Compared with traditional chemotherapy regimens, targeted therapy and immunotherapy have significantly improved the survival rates of advanced NSCLC patients (6–8). For patients with high PD-L1 expression, immune monotherapy can provide significant clinical benefits, with an objective response rate of approximately 45% and a 5-year survival rate as high as 32% (9, 10). This has thoroughly revolutionized the treatment paradigm for advanced lung cancer. However, the clinical benefits of immune monotherapy are not significant in populations with low or no PD-L1 expression. Patients with PD-L1 expression ≥50% account for only 29.8% of the total NSCLC population (11). The tissue samples required for PD-L1 immunohistochemical testing in lung cancer are difficult to obtain, and overcoming the spatiotemporal heterogeneity of tumors is challenging. Peripheral blood biomarkers, as supplements to tissue testing, offer the advantages of convenient sampling, noninvasiveness, and the ability for repeated sampling while also encompassing information from both tumor and host immune status.

Programmed cell death protein 1 (PD-1) is a membrane receptor protein that is present mainly on the surface of immune cells, such as activated T cells, B cells, and NK cells (12). Programmed death-ligand 1 (PD-L1) is the ligand protein of PD-1, a type I transmembrane protein with a size of 40 kDa that is present on the surface of tumor cells and certain antigen-presenting cells (13). PD-L1 on the surface of tumors can bind to PD-1 on the surface of activated T lymphocytes, thereby reducing the activity of T lymphocytes and inhibiting lymphocyte proliferation through the PD-1/PD-L1 pathway, thus suppressing the role of T lymphocytes in the local tumor microenvironment, reducing the immune killing function of tumors, and making tumors prone to immune escape (14).

Lymphocyte immunoglobulin-like molecule 3 (TIM-3) is expressed in T cells, macrophages, and dendritic cells and primarily performs inhibitory receptor functions. TIM-3 in the tumor microenvironment can exert immunosuppressive effects by inhibiting Th1 and Th17 cells, inducing exhaustion of CD8+ T cells, promoting the expansion of highly immunosuppressive Treg cell populations, facilitating massive expansion of bone marrow-derived suppressor cells (MDSCs) with potent T-cell immunosuppressive functions, and promoting innate immune suppression and tumor immune escape pathways (14). TIM-3 is expressed in various types of cancers, including osteosarcoma, cervical cancer, melanoma, lung cancer, etc., and its expression is associated with poor prognosis (14).

Unlike αβ T cells, which primarily recognize peptide antigens presented by major histocompatibility complex (MHC) molecules, γδ T cells utilize a different T-cell receptor (TCR) structure composed of γ and δ chains to recognize a wider range of antigens in an MHC-independent manner (15). As MHC-unrestricted innate-like lymphocytes, they bridge the innate and adaptive immune systems. γδ T cells can rapidly respond to various pathogens through their innate-like receptors, even in the absence of prior antigen exposure, enabling early immune defense (16). Based on the expression of the γ and δ chains in their TCRs, γδ T cells are classified into three subtypes: Vδ1+ T, Vδ2+ T, and Vδ3+ T cells (17). Among them, the Vδ2+ type functions primarily in lysing tumor cells. Vδ2+ T cells can be activated by phosphoantigens that accumulate in tumor cells, and the Vγ9Vδ2 TCR can also interact with proteins aberrantly upregulated in cancer cells (18). In addition to TCR-mediated antigen recognition, NKR also plays a crucial role in activating Vδ2+ T cells and initiating tumor lysis (19). Activated Vδ2+ T cells exert antitumor effects through multiple mechanisms, including but not limited to direct killing of tumor cells via NK cell receptors, induction of tumor cell apoptosis through apoptosis-related ligands (TRAIL/FASL), lysis of tumor cells through secreted granzyme/perforin, and killing of tumor cells through the antibody-dependent cell-mediated cytotoxicity (ADCC) effect. γδ T cells, which are positioned at the forefront of cancer immune surveillance, can detect the early transformation of cells into tumor cells (15).

Previously, researchers reported that, in studies of NSCLC patients treated with PD-1 inhibitors, an increase in peripheral blood CD8+ T cells predicts a good treatment response, whereas an increase in the total T-cell count and the CD4+/CD8+ T-cell ratio is significantly associated with a poorer response (20). Other researchers have confirmed that an increase in senescent T cells is associated with a poor prognosis in NSCLC patients treated with PD-1/PD-L1 inhibitors (21). Based on prior research, peripheral blood immune cells in NSCLC patients treated with anti-PD-1 therapy undergo varying degrees of change, and different subpopulations may exert distinct functions regardless of treatment efficacy. Therefore, the levels of indicators related to peripheral blood lymphocyte subsets are correlated with the efficacy of PD-1 inhibitor therapy and hold potential for predicting treatment outcomes. Building on previous studies, the present study aims to further identify response biomarkers to predict the efficacy of combination chemotherapy and PD-1 inhibitor therapy in NSCLC patients.

## Materials and methods

### Patients

This study was conducted at the Department of Oncology (Immunology) at the Fourth Hospital of Hebei Medical University and was approved by the Ethics Committee of the Fourth Hospital of Hebei Medical University. All individual participants provided informed consent.

Participants were enrolled in the study from March 2022 to June 2023 and met the following criteria: 1) all included non-small cell lung cancer patients received chemotherapy combined with anti-PD-1 treatment; 2) patients were at least 18 years old; 3) histologically or cytologically confirmed stage III or IV non-small cell lung cancer; and 4) fresh peripheral blood samples were collected immediately before receiving anti-PD-1 treatment. Healthy controls were individuals confirmed to be in good physical health through routine medical examinations, without any serious organic or functional diseases.

**Figure 1:**
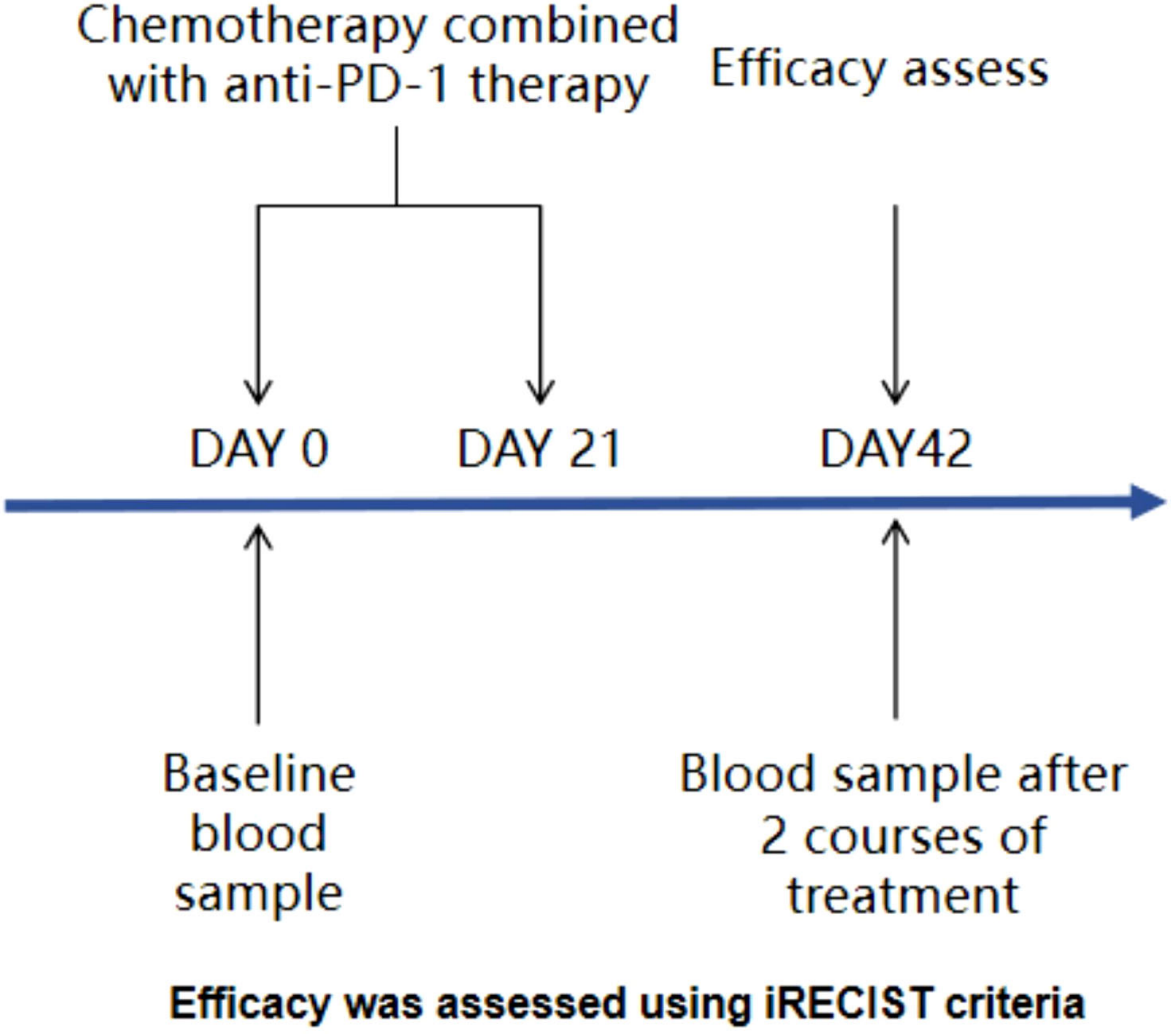
Study design.

After patient enrollment, blood samples were collected for immune cell and cytokine testing prior to treatment to establish baseline data. Following two cycles of chemotherapy combined with PD-1 inhibitor therapy, blood samples were again collected for immune cell and cytokine analysis as post-treatment data, concurrently with efficacy evaluation (**Figure 1**). The efficacy evaluation adhered to the iRECIST criteria: the R group comprised patients achieving partial response (PR) and complete response (CR). CR was defined as the disappearance of all target lesions, no emergence of new lesions, and normalization of tumor markers, sustained for at least 4 weeks. PR was defined as a reduction of ≥30% in the sum of the diameters of target lesions, lasting for at least 4 weeks. The NR group included patients with progressive disease (PD) and stable disease (SD). SD was defined as a reduction in the sum of the diameters of target lesions that did not meet the PR criteria or an increase that did not reach the PD threshold. PD was characterized by an increase of at least ≥20% in the sum of the diameters of target lesions or the appearance of new lesions. Chemotherapy and PD-1 inhibitor were administered synchronously, with a treatment cycle defined as once every 21 days. Chemotherapy regimens encompassed the AP (pemetrexed plus cisplatin) regimen, GP (gemcitabine plus cisplatin) regimen, and TP (paclitaxel-based chemotherapy plus cisplatin) regimen. PD-1 inhibitors included tislelizumab and sintilimab. The specific treatment plan was tailored by physicians based on the actual condition of the patients.

### Flow cytometry analysis

**Figure 2:**
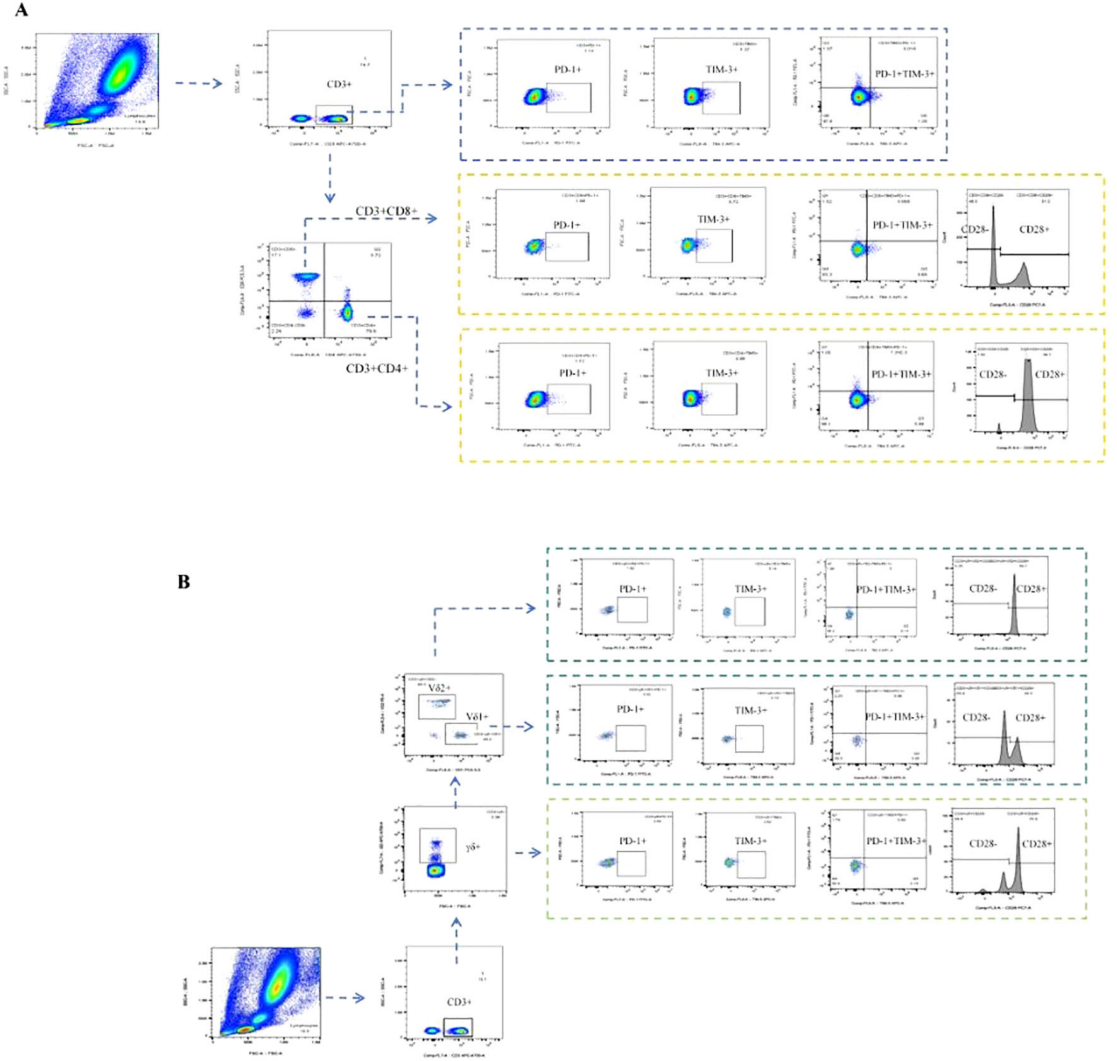
T-cell subpopulations were determined via flow cytometry. **(A)** Gating strategy for aβT cell subpopulations in flow cytometry: Gate CD3+ T cells, CD3+CD4+ T cells, CD3+CD8+ T cells, CD3+CD4-CD8- T cells, CD3+PD-1+ T cells, CD3+TIM-3+ T cells, CD3+TIM-3+PD-1+ T cells, CD3+CD4+PD-1+ T cells, CD3+CD4+TIM-3+ T cells, CD3+CD4+TIM-3+PD-1+ T cells, CD3+CD4+CD28+ T cells, CD3+CD4+CD28- T cells, CD3+CD8+PD-1+ T cells, CD3+CD8+TIM-3+ T cells, CD3+CD8+TIM-3+PD-1+ T cells, CD3+CD8+CD28+ T cells, CD3+CD8+CD28- T cells, separately. **(B)** Gating strategy for γδT cell subpopulations in flow cytometry: Gate CD3+γδT+ T cells, CD3+γδT+V81+ T cells, CD3+γδT+Vδ2+ T cells, CD3+γδT+PD-1+ T cells, CD3+γδT+TIM-3+ T cells, CD3+γδT+TIM-3+PD-1+ T cells, CD3+γδT+CD28+ T cells, CD3+γδT+CD28- T cells, CD3+γδT+Vδ1+PD-1+ T cells, CD3+γδT+Vδ1+TIM-3+ T cells, CD3+γδT+Vδ1+TIM-3+PD-1+ T cells, CD3+γδT+Vδ1+CD28+ T cells, CD3+γδT+Vδ1+CD28- T cells, CD3+γδT+Vδ2+PD-1+ T cells, CD3+γδT+Vδ2+TIM-3+ T cells, CD3+γδT+Vδ2+TIM-3+PD-1+ T cells, CD3+γδT+Vδ2+CD28+ T cells, CD3+γδT+Vδ2+CD28- T cells, separately.

The antibody MIX was first prepared for the experiment. Panel 1 included PerCP-Vio700 anti-human Vδ1 (clone: REA173, cat No: 130-120-441, Miltenyi), phycoerythrin (PE) anti-human Vδ2 (clone: B6, cat No: 331408, Biolegend), APC-H7 anti-human CD3 (clone: SK7, cat No: 560176, BD Biosciences), PE-Cy7 anti-human CD28 (clone: CD28.2, cat No: 302926, Biolegend), R718 anti-human γδ TCR (clone: 11F2, cat No: 752023, BD Biosciences), FITC anti-human CD279 (programmed cell death protein 1, PD-1) (clone: EH12.2H7, cat No: 329904, Biolegend), Alexa Fluor 647 anti-human CD366 (T-cell immunoglobulin domain and mucin domain-3, TIM-3) (clone: 7D3, cat No: 565558, BD Biosciences); Panel 2 included Alexa Fluor 700 anti-human CD3 (clone: OKT3, cat No: 317340, Biolegend), APC-Cy7 anti-human CD4 (clone: RPA-T4, cat No: 557871, BD Biosciences), PerCP-Cy5.5 anti-human CD8 (clone: SK1, cat No: 565310, BD Biosciences), PE-Cy7 anti-human CD28 (clone: CD28.2, cat No: 302926, Biolegend), FITC anti-human CD279 (programmed cell death protein 1, PD-1) (clone: EH12.2H7, cat No: 329904, Biolegend), and Alexa Fluor 647 anti-human CD366 (T cell immunoglobulin domain and mucin domain-3, TIM-3) (clone: 7D3, cat No: 565558, BD Biosciences). Then, 100 μL of venous blood and 10 μL of the MIX antibody were added to the flow tubes, which were shaken gently and incubated in the dark for 15 min. After incubation, 800 μL of red blood cell lysis buffer (Solarbio, Beijing, China) was added to the tubes for lysis at room temperature for 15 min. After lysis, 2 mL of phosphate-buffered saline (PBS, Solarbio, Beijing, China) was added to stop the lysis, and the tubes were shaken gently and centrifuged at 1000 rpm for 5 min. Finally, the resulting cell pellets were resuspended in 350 μL of PBS. The fluorescence of different cell subsets was detected via BD LSRFortessa flow cytometry. The gating strategy is shown in **Figure 2**.

### ELISA

The levels of IFN-γ (JL12152), TNF‐α (JL10208), TGF-β (JL20082), IL-1α (JL48533), IL-1β (JL13662), IL-2 (JL19265), IL-4 (JL19287), IL‐6 (JL14113), IL-8 (JL19291), IL-17 (JL19255), CXCL9 (JL14160), CXCL10 (JL11028), CXCL11 (JL11358), and CXCL13 (JL10292) in blood serum were measured via ELISA kits according to the manufacturer’s instructions.

### Statistical analysis

The Mann–Whitney U test was performed to determine significant differences between two groups. Paired samples were compared via the Wilcoxon matched-pairs signed-rank test. Categorical variables are summarized as numbers and percentages and were compared via the chi-square test or Fisher’s exact test. The optimal model parameters were selected through binomial logistic regression analysis using a forward stepwise regression approach based on maximum likelihood estimation. The model was internally validated using the leave-one-out cross-validation (LOOCV) method, and a receiver operating characteristic (ROC) curve was plotted to assess the model’s discriminative ability. The cutoff points were calculated via the maximum Youden index. The Hosmer-Lemeshow goodness-of-fit test was employed to evaluate the model’s calibration. All tests were two-sided; significance levels were set to P < 0.05 (*), P < 0.01 (**), P < 0.001 (***), and P < 0.0001 (****), and NS means not significant. Data statistics and data visualization were implemented using GraphPad Prism version 8.0, SPSS version 25.0 software, and R version 4.4.1.

## Results

A total of 32 patients with NSCLC were treated with chemotherapy combined with anti-PD-1 therapy. Overall, 22 patients achieved partial response (PR), 7 patients had stable disease (SD), and 3 patients had progressive disease (PD), which led to an objective response ratio of 62.9% (22/35). The detailed patient characteristics are shown in **Table 1**. The median age of the entire cohort was 63.5 years (range 46–75), and 93.8% (30/32) of the patients were male. A smoking history was identified in 81.3% (26/32) of the patients. Adverse effects were found in 71.9% (23/32) of the patients. The level of total bilirubin (T-bil) was significantly higher in the CR/PR group compared to the SD/PD group (p = 0.025).

**Table 1:** Patients’ characteristics.

Characteristics	Total	CR/PR	SD/PD	p Value
	(n=32)	(n=22)	(n=10)	
Age, years, median (range)	63.5 (46-75)	62.5 (46-75)	65 (53-75)	0.425
<60	11 (34.4)	9 (40.9)	2 (20.0)	
≥60	21 (65.6)	13 (59.1)	8 (80.0)	
Gender				0.534
Male	30 (93.8)	21 (95.5)	9 (90.0)	
Female	2 (6.3)	1 (4.5)	1 (10.0)	>0.999
Pathological type				
Squamous cell carcinoma	23 (71.9)	16 (72.7)	7 (70.0)	
Adenocarcinoma	9 (28.1)	6 (27.3)	3 (30.0)	
Smoking history				0.637
Yes	26 (81.3)	17 (77.3)	9 (90.0)	
No	6 (18.7)	5 (22.7)	1 (10.0)	
Adverse effects				0.407
Yes	23 (71.9)	17 (53.1)	6 (60.0)	
No	9 (28.17)	5 (46.9)	4 (40.0)	
PD-L1 (TPS)				0.855
TPS>50%	4 (12.5)	3 (13.6)	1 (10.0)	
1%<TPS ≤ 50%	6 (18.8)	4 (18.2)	1 (10.0)	
TPS ≤ 1%	22 (68.7)	15 (68.2)	7 (70.0)	
NA	0 (0.0)	0(0.0)	1(10.0)	
SCCA, ng/mL, median (range)	2.94 (0.63-26.07)	2.99 (0.63-26.07)	2.53 (1.18-20.02)	0.593
ProGRP, pg/mL, median (range)	43.82 (23.41-95.10)	42.36 (23.41-95.10)	44.91 (37.31-83.80)	0.326
CEA, ng/mL, median (range)	4.08 (1.04-889.20)	3.51 (1.04-889.20)	4.25 (2.07-115.80)	0.379
CYFRA21-1, ng/mL, median (range)	7.39 (2.26-106.50)	6.67 (2.26-106.50)	9.08 (2.79-64.06)	0.616
NSE, ng/mL, median (range)	17.00 (7.95-86.38)	16.27 (7.95-34.65)	18.90 (13.74-86.38)	0.160
Height, cm, median (range)	170 (150-183)	170 (150-183)	170 (155-175)	0.754
Weight, Kg, median (range)	65.0 (45.0-85.0)	65.0 (45.0-85.0)	62.0 (55.0-82.0)	0.789
BMI, median (range)	22.77 (16.71-26.83)	22.53 (16.71-26.83)	22.89 (18.12-26.78)	0.541
WBC, 10E9/L, median (range)	7.13 (4.05-14.78)	6.96 (4.22-14.32)	7.26 (4.05-14.78)	0.949
Neutrophils, 10E9/L, median (range)	4.48 (1.36-12.87)	4.67 (3.05-12.07)	4.29 (1.36-12.87)	0.848
Lymphocytes, 10E9/L, median (range)	1.37 (0.58-2.46)	1.53 (0.58-2.46)	1.36 (1.03-2.42)	0.616
NLR, median (range)	3.69 (1.00-12.42)	3.79 (1.56-10.71)	3.60 (1.00-12.42)	0.716
Monocytes, 10E9/L, median (range)	0.52 (0.07-1.02)	0.56 (0.07-1.02)	0.47 (0.32-0.72)	0.873
Eosinophils, 10E9/L, median (range)	0.13 (0.01-0.41)	0.15 (0.02-0.41)	0.09 (0.01-0.34)	0.126
Basophils, 10E9/L, median (range)	0.02 (0.00-0.07)	0.03 (0.00-0.07)	0.01 (0.01-0.05)	0.276
RBC, 10E12/L, median (range)	4.32 (3.62-5.34)	4.31 (3.62-5.17)	4.39 (3.79-5.34)	0.873
HGB, g/L, median (range)	128.00 (106.00-170.00)	131.50 (106.00-170.00)	126.00 (106.00-152.00)	0.756
Platelets, 10E9/L, median (range)	294.00 (188.00-638.00)	293.50 (213.00-638.00)	297.00 (188.00-418.00)	0.741
Sodium, mmol/L, median (range)	138.00 (131.00-141.00)	137.50 (131.00-141.00)	138.00 (131.00-141.00)	0.801
Potassium, mmol/L, median (range)	4.50 (3.80-5.50)	4.45 (3.80-5.50)	4.50 (3.80-4.70)	0.707
Calcium, mmol/L, median (range)	2.36 (2.20-2.79)	2.37 (2.24-2.54)	2.35 (2.20-2.79)	0.873
ALT, U/L, median (range)	24.10 (5.70-56.00)	24.05 (10.50-56.00)	24.80 (5.70-38.10)	0.453
AST, U/L, median (range)	19.90 (13.50-31.70)	20.50 (13.50-31.70)	18.10 (14.80-24.90)	0.277
ALB, g/L, median (range)	39.10 (17.20-45.10)	39.95 (17.20-45.10)	37.20 (34.80-42.10)	0.646
T-bil, μmol/L, median (range)	6.80 (3.70-20.70)	7.05 (3.70-20.70)	5.80 (5.20-11.70)	0.025*
D-bil, μmol/L, median (range)	3.70 (0.20-5.80)	3.80 (0.70-5.80)	3.20 (0.20-5.60)	0.083
I-bil, μmol/L, median (range)	3.40 (0.90-15.40)	4.40 (0.90-15.40)	2.60 (2.00-6.10)	0.121
CRE, μmol/L, median (range)	59.10 (35.00-115.60)	61.20 (42.70-115.60)	53.50 (35.00-71.70)	0.104
UREA, mmol/L, median (range)	5.30 (2.40-14.50)	5.10 (2.70-13.10)	6.30 (2.40-14.50)	0.585
LDH, U/L, median (range)	195.00 (110.00-499.00)	186.50 (110.00-297.00)	213.00 (156.00-499.00)	0.199

CR, complete response; PR, partial response; SD, stable disease; PD, progressive disease; PD-L1, programmed death-ligand 1; TPS, tumor proportion score; SCCA, squamous cell carcinoma antigen; ProGRP, pro-gastrin-releasing peptide; CEA, carcinoembryonic antigen; CYFRA21-1, cytokeratin 19 fragment; NSE, neuron-specific enolase; BMI, body mass index; WBC, white blood cell; NLR, neutrophil-to-lymphocyte ratio; RBC, red blood cell; HGB, hemoglobin; ALT, alanine transaminase, AST, Aspartate aminotransferase; ALB, albumin; T-bil, total bilirubin; D-bil, direct bilirubin; I-bil, indirect bilirubin; CRE, creatinine; LDH, lactate dehydrogenase.

p Values were estimated by Fisher’s exact test and the Mann–Whitney U test for categorical variables and continuous variables, respectively.

*P < 0.05.

**Figure 3:**
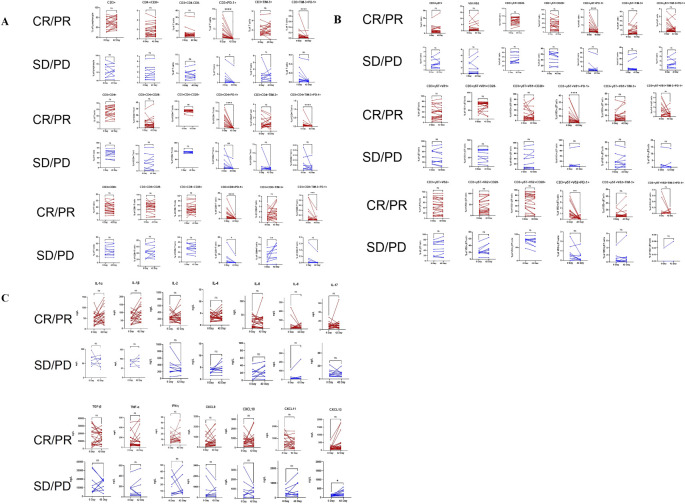
Pairwise comparisons between pre- and post-anti-PD-1 plus chemotherapy treatment in the CR/PR and SD/PD groups for changes in T lymphocyte subsets and cytokines via the Wilcoxon matched-pairs signed-rank test. **(A)**: Changes in aβT cells in the CR/PR and SD/PD groups. **(B)**: Changes in γδT cells in the CR/PR and SD/PD groups. **(C)**: Changes in cytokine levels in the CR/PR and SD/PD groups. CR, complete response; PR, partial response; SD, stable disease; PD, progressive disease. ^ns^P ≥ 0.05, *P < 0.05, **P < 0.01, ***P < 0.001, ****P < 0.0001.

The lymphocyte subset circular logic of T cells is shown in **Figure 2**. **Table 2** presents the baseline percentages of T lymphocyte subsets in the CR/PR and SD/PD groups. In the CR/PR group, the proportion of CD3+γδT+CD28- T cells was significantly higher compared to that in the SD/PD group (p=0.039, p=0.055). **Figure 3** compares the lymphocyte subset and cytokine data after 6 weeks of combination chemotherapy and anti-PD-1 therapy in both the CR/PR and SD/PD groups. In the CR/PR group, the proportions of CD3+TIM-3+PD-1+, CD3+CD4+TIM-3+PD-1+, CD3+CD8+TIM-3+PD-1+, CD3+γδT+PD-1+, CD3+γδT+Vδ1+PD-1+, and CD3+γδT+Vδ2+PD-1+ T cells significantly decreased after 6 weeks of chemotherapy combined with anti-PD-1 therapy (p<0.0001, p<0.001, p<0.0001, p<0.001, p<0.05), whereas no significant differences were detected in the SD/PD group (p>0.05).

**Table 2:** Characteristics of baseline lymphocyte subpopulations and cytokines.

	Total	CR/PR	SD/PD	p Value
	(n=32)	(n=22)	(n=10)	
CD3+, % of lymphocyte, median (range)	67.70 (42.30-89.80)	67.90 (50.70-87.10)	62.85 (42.30-89.80)	0.483
CD3+CD4+, % of T cells, median (range)	52.95 (22.30-78.40)	52.30 (22.30-72.60)	57.75 (34.10-78.40)	0.515
CD3+CD8+, % of T cells, median (range)	40.65 (12.80-68.30)	41.20 (12.80-68.30)	37.30 (17.50-63.40)	0.704
CD4+/CD8+, median (range)	1.32 (0.38-5.41)	1.25 (0.38-5.41)	1.57 (0.54-4.48)	0.652
CD3+CD4-CD8-, % of T cells, median (range)	4.23 (1.17-32.30)	4.23 (1.17-32.30)	4.61 (1.21-8.60)	0.646
CD3+CD4+CD28-, % of CD4+T cells, median (range)	4.90 (0.31-29.80)	5.23 (0.31-29.80)	4.46 (0.45-13.90)	0.458
CD3+CD4+CD28+, % of CD4+T cells, median (range)	95.10 (70.20-99.70)	94.80 (70.20-99.70)	95.55 (86.10-99.50)	0.464
CD3+CD8+PD-1+, % of CD8+T cells, median (range)	1.56 (0.10-24.00)	1.59 (0.31-15.60)	1.32 (0.10-24.00)	0.882
CD3+CD8+CD28-, % of CD8+T cells, median (range)	53.15 (8.13-86.10)	55.60 (8.13-86.10)	45.90 (21.60-69.70)	0.137
CD3+CD8+CD28+, % of CD8+T cells, median (range)	46.85 (13.90-91.90)	44.40 (13.90-91.90)	54.10 (30.30-78.40)	0.137
CD3+PD-1+, % of T cells, median (range)	1.98 (0.12-13.60)	2.15 (0.62-12.10)	1.36 (0.12-13.60)	0.305
CD3+CD4+PD-1+, % of CD4+T cells, median (range)	2.56 (0.18-12.10)	3.07 (0.84-12.10)	1.66 (0.18-7.94)	0.100
CD3+TIM-3+, % of T cells, median (range)	1.47 (0.27-7.50)	1.47 (0.83-5.27)	1.50 (0.27-7.50)	0.952
CD3+CD4+TIM-3+, % of CD4+T cells, median (range)	0.82 (0.40-13.30)	0.89 (0.40-3.15)	0.76 (0.45-13.30)	0.412
CD3+CD8+TIM-3+, % of CD8+T cells, median (range)	2.24 (0.03-6.81)	2.22 (0.04-6.81)	2.53 (0.03-4.97)	0.764
CD3+TIM-3+PD-1+, % of T cells, median (range)	0.06 (0.00-0.88)	0.07 (0.01-0.88)	0.06 (0.00-0.54)	0.623
CD3+CD4+TIM-3+PD-1+, % of CD4+T cells, median (range)	0.05 (0.00-0.98)	0.06 (0.00-0.98)	0.05 (0.00-0.23)	0.254
CD3+CD8+TIM-3+PD-1+, % of CD8+T cells, median (range)	0.05 (0.00-1.57)	0.05 (0.01-0.92)	0.04 (0.00-1.57)	0.992
CD3+γδT+, % of T cells, median (range)	2.75 (0.77-20.60)	3.29 (0.97-20.60)	2.29 (0.77-6.08)	0.154
CD3+γδT+Vδ1+, % of γδT cells, median (range)	37.30 (2.53-83.80)	37.55 (2.53-83.80)	31.55 (12.70-82.30)	0.833
CD3+γδT+Vδ2+, % of γδT cells, median (range)	48.00 (6.03-94.50)	47.45 (6.03-94.50)	53.40 (15.20-80.00)	>0.999
Vδ1+/Vδ2+, median (range)	0.77 (0.03-12.60)	0.77 (0.03-12.60)	0.61 (0.16-5.41)	0.865
CD3+γδT+Vδ1+PD-1+, % of Vδ1+γδT cells, median (range)	4.33 (0.00-70.20)	6.20 (0.76-36.60)	2.51 (0.00-70.20)	0.058
CD3+γδT+Vδ2+PD-1+, % of Vδ2+γδT cells, median (range)	0.87 (0.00-4.09)	0.92(0.00-4.09)	0.73 (0.00-2.52)	0.991
CD3+γδT+CD28-, % of γδT cells, median (range)	61.65 (16.00-95.80)	69.90 (16.00-95.80)	40.40 (29.80-86.00)	0.039*
CD3+γδT+CD28+, % of γδT cells, median (range)	38.35 (4.19-84.00)	30.10 (4.19-84.00)	59.60 (14.00-70.20)	0.039*
CD3+γδT+Vδ1+CD28-, % of Vδ1+γδT cells, median (range)	85.75 (12.40-99.10)	86.30 (21.20-99.10)	71.40 (12.40-98.50)	0.741
CD3+γδT+Vδ1+CD28+, % of Vδ1+γδT cells, median (range)	14.25 (0.89-87.60)	13.70 (0.89-78.80)	28.60 (1.48-87.60)	0.764
CD3+γδT+Vδ2+CD28-, % of Vδ2+γδT cells, median (range)	24.40 (4.08-97.90)	29.60 (4.08-97.90)	20.35 (5.19-52.90)	0.305
CD3+γδT+Vδ2+CD28+, % of Vδ2+γδT cells, median (range)	75.60 (2.06-95.90)	70.40 (2.06-95.90)	79.65 (47.10-94.80)	0.305
CD3+γδT+PD-1+, % of γδT cells, median (range)	2.09 (0.49-21.50)	2.33 (0.49-13.20)	1.51 (0.66-21.50)	0.509
CD3+γδT+TIM-3+, % of γδT cells, median (range)	4.35 (0.66-24.30)	4.30 (0.66-24.30)	4.35 (1.84-8.39)	0.617
CD3+γδT+Vδ1+TIM-3+, % of Vδ1+γδT cells, median (range)	5.29 (0.00-38.90)	4.92 (0.00-38.90)	5.29 (0.00-18.10)	0.682
CD3+γδT+Vδ2+TIM-3+, % of Vδ2+γδT cells, median (range)	1.26 (0.00-31.50)	2.38 (0.00-31.50)	0.57 (0.00-4.80)	0.050
CD3+γδT+TIM-3+PD-1+, % of γδT cells, median (range)	0.20 (0.00-4.14)	0.18 (0.00-2.66)	0.28 (0.00-4.14)	0.568
CD3+γδT+Vδ1+TIM-3+PD-1+, % of Vδ1+γδT cells, median (range)	0.36 (0.00-15.10)	0.43 (0.00-4.35)	0.31 (0.00-15.10)	0.976
CD3+γδT+Vδ2+TIM-3+PD-1+, % of Vδ2+γδT cells, median (range)	0.00 (0.00-0.74)	0.00 (0.00-0.74)	0.00 (0.00-0.04)	0.261
IL-1α, ng/L, median (range)	55.38 (12.54-264.37)	52.01 (12.54-264.37)	71.41 (40.06-234.09)	0.113
IL-1β, ng/L, median (range)	72.09 (21.71-730.26)	67.46 (21.71-730.26)	94.57 (60.62-431.47)	0.177
IL-2, ng/L, median (range)	237.70 (54.36-933.93)	235.85 (83.01-577.36)	329.87 (54.36-933.93)	0.459
IL-4, ng/L, median (range)	3.44 (1.35-6.68)	3.35 (1.35-6.68)	4.11 (1.57-4.93)	0.693
IL-6, ng/L, median (range)	23.99 (1.66-57.73)	26.43 (1.66-57.73)	16.08 (1.74-44.49)	0.356
IL-8, ng/L, median (range)	12.80 (5.59-241.92)	11.38 (5.59-241.92)	18.78 (9.01-110.87)	0.269
IL-17, ng/L, median (range)	7.29 (2.29-28.23)	7.69 (2.29-28.23)	7.05 (3.89-21.77)	0.915
TNF-α, ng/L, median (range)	68.31 (23.22-509.98)	62.45 (23.22-509.98)	155.01 (24.05-478.84)	0.678
TGF-β, ng/L, median (range)	12279.76 (1739.04-36130.78)	16614.69 (1739.04-36130.78)	10288.13 (4235.48-33822.40)	0.593
IFN-γ, ng/L, median (range)	10.79 (3.09-135.19)	10.36 (3.09-135.19)	16.77 (3.40-42.74)	0.564
CXCL9, ng/L, median (range)	293.06 (26.76-2754.33)	307.75 (26.76-2195.68)	184.62 (75.85-2754.33)	0.564
CXCL10, ng/L, median (range)	436.07 (91.29-3332.66)	507.80 (104.89-2296.38)	423.69 (91.29-3332.66)	0.848
CXCL11, ng/L, median (range)	504.98 (61.50-1779.60)	607.23 (61.50-1779.60)	266.83 (103.03-912.67)	0.078
CXCL13, ng/L, median (range)	87.57 (38.14-666.22)	128.46 (38.14-666.22)	80.43 (42.49-189.37)	0.188

p Values were estimated by the Mann–Whitney U test.

*P < 0.05.

**Figure 4:**
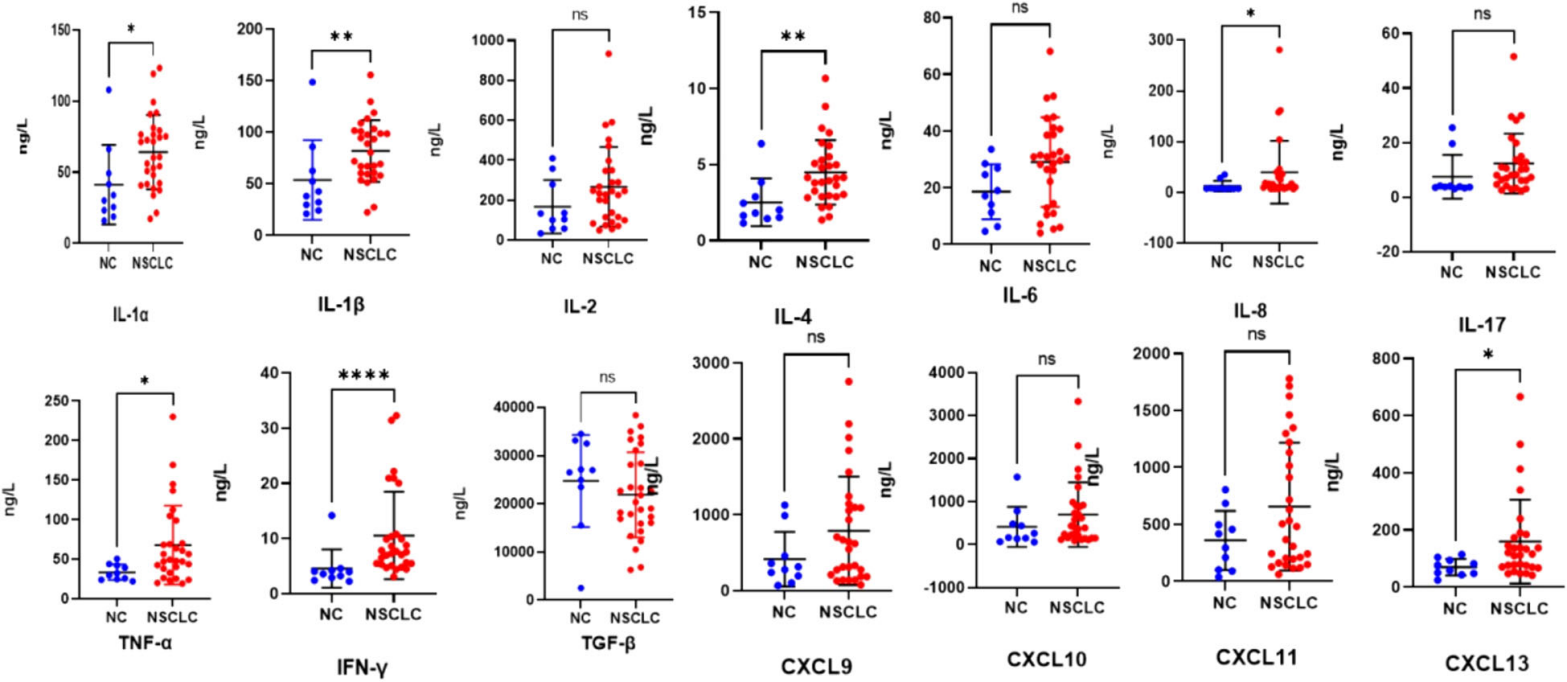
Scatter plots (means ± SDs) of baseline cytokine signatures between the NC and NSCLC groups. NC, normal control; NSCLC, non-small cell lung cancer. ^ns^P ≥ 0.05, *P < 0.05, **P < 0.01, ****P < 0.0001.

At baseline, the levels of certain cytokines in the peripheral blood of patients in the NSCLC group, encompassing both the CR/PR and SD/PD subgroups, exhibited differences compared to those in healthy controls. The concentrations of IL-1α, IL-1β, IL-4, IL-8, TNF-α, IFN-γ, and CXCL13 were significantly elevated compared to those in healthy controls, with the results demonstrating statistically significant differences (p < 0.05, p < 0.01, p < 0.01, p < 0.05, p < 0.05, p < 0.0001, and p < 0.05). (**Figure 4**). There was no significant difference in the contents of IL-2, IL-6, IL-17, TGF-β, CXCL10, or CXCL11 (p > 0.05) (**Figure 4**).

Peripheral indicators combined with clinically relevant information can, to some extent, predict the early treatment response in advanced NSCLC. Clinical laboratory data and immune data at baseline (pre-treatment) were analyzed for the CR/PR and SD/PD groups (**Tables 1**, **2**). Significantly different indicators, including T-bil (total bilirubin) (p=0.025), the percentage of CD3+γδT+Vδ2+TIM-3+ T cells (P=0.050), the percentage of CD3+γδT+CD28- T cells (p=0.039), and clinically relevant indicators such as age, gender, smoking history, tumor type, TPS (PD-L1), and the percentage of CD3+PD-1+ T cells, were selected as candidate indicators for model development. Collinearity analysis of these candidate indicators (**Table 3**) revealed that the tolerance for all indicators was well above 0.1, and the variance inflation factors were all below 10, indicating no collinearity.

**Table 3:** Results of collinearity analysis.

Covariates	Collinearity Tolerance	Statistics VIF
Gender	0.472	2.120
Age	0.758	1.319
Pathological type	0.855	1.170
Smoking history	0.413	2.420
CD3+γδT+Vδ2+TIM-3+	0.534	1.874
CD3+γδT+CD28-	0.721	1.387
CD3+PD-1+	0.808	1.238
T-bil	0.833	1.200
TPS	0.609	1.643

Dependent Variable: efficacy evaluation.

Using forward stepwise regression based on maximum likelihood estimation, a binomial logistic regression model was derived for the candidate indicators (**Table 4**). The relevant variables ultimately included in the binomial multifactorial regression model were Pathological type, Smoking history, the percentage of CD3+γδT+CD28- T cells, the percentage of CD3+PD-1+ T cells, and T-bil. The model formula is: ln(P/1-P) = -45.871 - 4.127 × Pathological type - 17.666 × Smoking history + 0.527 × the percentage of CD3+γδT+CD28- T cells + 4.604 × the percentage of CD3+PD-1+ T cells + 3.131 × T-bil. Here, P represents the probability of CR/PR occurrence, and 1-P represents the probability of SD/PD occurrence. Overall, the model was statistically significant (p < 0.05).

**Table 4:** Stepwise method for establishing a binomial logistic regression prediction model.

		B	S.E.	Wald	df	Sig.	Exp(B)	95%C.I. for EXP(B)	
								Lower	Upper
Step 1^a^	T-bil	0.287	0.189	2.321	1	0.128	1.333	.921	1.929
	Constant	-1.250	1.341	0.869	1	0.351	0.286		
Step 2^b^	CD3+γδT+CD28-	0.044	0.023	3.776	1	0.052	1.045	1.000	1.092
	T-bil	.372	0.212	3.062	1	0.080	1.450	0.956	2.198
	Constant	-4.476	2.272	3.883	1	0.049	0.011		
Step 3^c^	CD3+γδT+CD28-	0.084	0.040	4.369	1	0.037	1.088	1.005	1.177
	CD3+PD-1+	0.674	0.368	3.356	1	0.067	1.962	0.954	4.035
	T-bil	0.475	0.241	3.890	1	0.049	1.607	1.003	2.576
	Constant	-9.128	4.183	4.761	1	0.029	0.000		
Step 4^d^	Smoking history	-10.189	5.013	4.131	1	0.042	0.000	0.000	0.695
	CD3+γδT+CD28-	0.324	0.145	4.970	1	0.026	1.382	1.040	1.837
	CD3+PD-1+	2.914	1.352	4.648	1	0.031	18.429	1.303	260.600
	T-bil	1.851	0.830	4.967	1	0.026	6.365	1.250	32.407
	Constant	-28.883	13.163	4.815	1	0.028	0.000		
Step 5^e^	Pathological type	-4.127	2.995	1.899	1	0.168	0.016	0.000	5.711
	Smoking history	-17.666	15.515	1.296	1	0.255	0.000	0.000	342195.500
	CD3+γδT+CD28-	0.527	0.269	3.824	1	0.051	1.694	0.999	2.872
	CD3+PD-1+	4.604	2.453	3.523	1	0.061	99.865	0.816	12224.622
	T-bil	3.131	1.634	3.671	1	0.055	22.887	0.930	562.951
	Constant	-45.871	26.719	2.947	1	0.086	0.000		

a. Variable(s) entered on step 1: Tbil.

b. Variable(s) entered on step 2: CD3+γδT+CD28-.

c. Variable(s) entered on step 3: CD3+PD-1+.

d. Variable(s) entered on step 4: Smoking history.

e. Variable(s) entered on step 5: Pathological type.

f. Stepwise procedure stopped because removing the least significant variable results in a previously fitted model.

**Figure 5:**
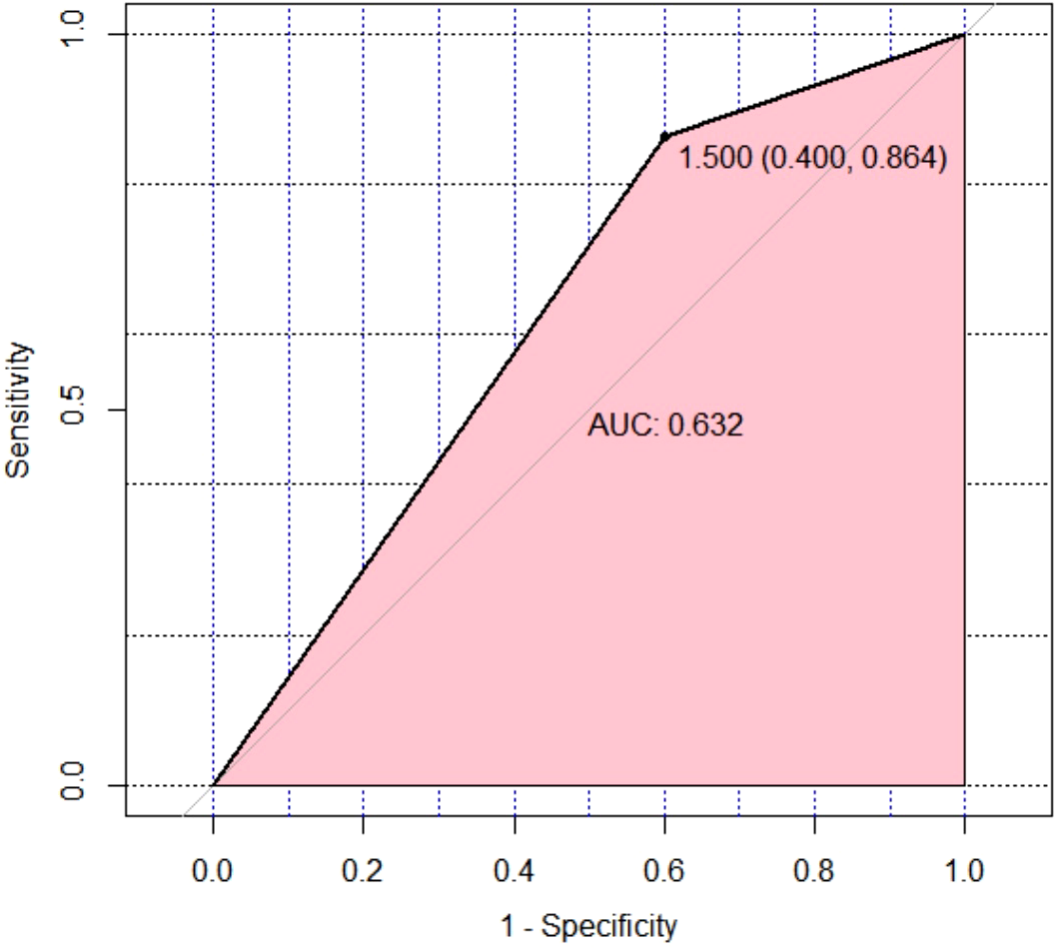
Leave-One-Out Cross-Validation was performed on the multivariate binomial logistic regression model for therapeutic effect prediction, and an ROC curve was plotted to visually assess the model’s discriminative ability. ROC, receiver operating characteristic.

The Hosmer-Lemeshow goodness-of-fit test result was not significant (P=0.952), indicating that the information in the current data has been fully extracted, and the model has a good fit (**Table 5**). Internal cross-validation of the model using the leave-one-out method yielded an accuracy of 0.656 and a Kappa coefficient of 0.178, suggesting that the model has some predictive ability but relatively low consistency. The performance of the model was visually presented using the ROC (Receiver Operating Characteristic) curve (**Figure 5**). The ROC curve is an effective tool for evaluating the performance of binary classification models, plotting the true positive rate (Sensitivity, or recall) against the false positive rate (1-Specificity) to intuitively reflect the model’s classification ability at different thresholds.

**Table 5:** Hosmer and lemeshow test.

Step	Chi-square	df	Sig.
1	12.996	8	0.112
2	12.327	8	0.137
3	12.308	8	0.138
4	2.518	8	0.961
5	2.693	8	0.952

From **Figure 5**, it can be observed that the ROC curve of the model exhibits a certain degree of discrimination, indicating that the model can distinguish between positive and negative classes to some extent. Specifically, the calculated area under the curve (AUC) was 0.632. The AUC value ranges between 0.5 (random guessing level) and 1.0 (perfect classifier), and an AUC of 0.632 indicates that the model has some predictive ability but still has room for improvement. Further analysis of the model’s sensitivity and specificity yielded a Sensitivity of 0.864 and a Specificity of 0.600. A high sensitivity implies that the model can well identify samples that are actually positive, i.e., a high true positive rate, which is a positive sign in this study, indicating strong recognition ability of the model for the target category. However, the relatively low specificity (0.600) suggests that the model has some misjudgments when identifying negative samples, i.e., a certain proportion of false positives occur.

## Discussion

PD-1 blockade therapy has been widely applied in NSCLC patients, significantly improving patient survival and prognosis (22). However, some patients exhibit poor response to PD-1 inhibitor therapy, and the underlying mechanism remain incompletely elucidated (23). Clinically, the tumor proportion score (TPS) serves as one of the important reference indicators for determining the suitability of NSCLC patients for PD-1 monoclonal antibody therapy. TPS represents the percentage of tumor cells expressing PD-L1 within the tumor tissue, reflecting the level of PD-L1 expression. PD-1 monoclonal antibodies exert their therapeutic effects by blocking the binding of PD-1 to PD-L1, thereby restoring the anti-tumor activity of T cells. The expression level of PD-L1 is a key factor influencing the efficacy of PD-1 monoclonal antibody therapy. Our study demonstrated that there was no significant difference in PD-L1 expression in tumor tissues between the group with a good response and the group with a poor response to chemotherapy combined with PD-1 inhibitor treatment (**Table 1**). Studies have shown that some NSCLC patients without PD-L1 expression may respond to anti-PD-1 drugs (24), and 50% of patients with high PD-L1 expression in tumors cannot benefit from first-line pembrolizumab (anti-PD-1 antibody) (9). When faced with unresectable tumors, obtaining accurate tissue samples for PD-L1 immunohistochemical detection becomes challenging, and overcoming the spatiotemporal heterogeneity of tumors is difficult (25). Moreover, a single target cannot accurately predict and comprehensively reflect the immune status of patients receiving anti-PD-1 immunotherapy. Our study examined immunosuppressive markers (including PD-1 and TIM-3) on peripheral blood T cells and their subsets (including αβT and γδT cells) in NSCLC patients receiving chemotherapy combined with anti-PD-1 therapy, and monitored the cytokine levels in the peripheral blood. The combination of key immune indicators in peripheral blood was used as a potential method to determine the efficacy of chemotherapy combined with anti-PD-1 immunotherapy for NSCLC.

Studies have shown that the cytotoxicity of tumor-infiltrating T cells is closely related to the cytotoxicity of peripheral T cells, so monitoring the activity of circulating T cells may reflect the immune response in the tumor microenvironment (26, 27). Compared with biopsy samples of tumor tissue, peripheral blood markers have the advantages of being easy to obtain, noninvasive, and repeatable, and they present less heterogeneity as a supplement to tissue testing. In this study, we detected peripheral immune cells in NSCLC patients before and after receiving chemotherapy combined with anti-PD-1 therapy. There was no significant difference in the levels of CD4+ T, CD8+ T, or γδ T cells in the peripheral blood of patients in the CR/PR or SD/PD groups before treatment, and some results are consistent with those of early studies (27–30). We found that the proportions of CD3+TIM-3+PD-1+, CD3+CD4+TIM-3+PD-1+, CD3+CD8+TIM-3+PD-1+, CD3+γδT+PD-1+, CD3+γδT+Vδ1+PD-1+ and CD3+γδT+Vδ2+PD-1+T cells in the CR/PR group decreased after 6 weeks of chemotherapy combined with anti-PD-1 therapy but did not change in the SD/PD group, indicating that changes in the proportions of T lymphocyte subsets may be related to the treatment response.

The inhibition of coinhibitory molecules is crucial for regulating immune responses and cancer progression (31). During anti-PD-1 immunotherapy, multiple immune checkpoint molecules expressed by T cells may undergo changes, reflecting alterations in immune function (32). Following anti-PD-1 therapy, there are also different changes in the expression of coinhibitory molecules on T cells (33). Studies have shown that high expression of PD-1 on peripheral blood CD4+ T cells in NSCLC patients receiving anti-PD-L1 therapy is associated with adverse clinical outcomes. Other studies have indicated that after anti-PD-1 therapy, TIM-3 expression on T cells is increased in PD NSCLC patients, whereas TIM-3 expression on T cells is decreased in the SD group (34). Our study revealed that in the group that responded well to chemotherapy combined with anti-PD-1 therapy, the coexpression of PD-1 and TIM-3 on peripheral blood CD3+, CD3+CD4+, and CD3+CD8+ T cells significantly decreased after 6 weeks of treatment, whereas there was no significant difference in the SD/PD group. Other studies have also reported elevated levels of related immune inhibitory molecules (PD-1, LAG3, TIM-3, and TIGIT) in the response group following PD-1 inhibitor therapy (35). Heterogeneity in the results may arise from different treatment regimens and monitoring during different treatment periods. We speculate that coinhibitory molecules may have the potential to predict the efficacy of immunotherapy in patients.

Cytokines functionally participate in cell growth, proliferation, survival, differentiation, migration, and immune activation. The cytokine profile transmitted by immune cells determines the immune response of cells. Increasing evidence suggests a link between chronic infection and inflammation and the occurrence of tumors. Local inflammation in the tumor microenvironment attracts various immune cells, including αβ T cells, γδ T cells, and natural killer (NK) T cells, all of which play important roles in tumor immunity (36). Our research revealed that, compared with those in healthy individuals, the levels of proinflammatory cytokines, including IL-1, IL-2, IL-8, IFN-γ, and TNF-α, which favorably regulate cell-mediated immunity and exert certain antitumor effects, are generally elevated in the serum of advanced NSCLC patients. IL-4 is an anti-inflammatory cytokine, and we detected a significant increase in serum IL-4 in NSCLC patients, which may contribute to the survival, growth, and metastasis of tumor cells. Studies have shown that marrow IL-4 signaling drives myeloid cell production, thereby promoting tumor progression (37).

The dynamic changes in circulating immune cell subsets, especially T lymphocyte subsets, can provide predictive value for the effectiveness of PD-1 inhibitor therapy. Several studies have focused on the predictive value of the CD8+PD-1+ T-cell proliferation response (27, 38). It has been reported that NSCLC patients with a greater proportion of highly differentiated CD4+CD27−CD28− T cells are more likely to achieve excellent clinical outcomes (39). Our study not only included αβ T cells but also investigated the changes in the levels of cell surface coinhibitory molecules (PD-1 and TIM-3) in major subsets of γδ T cells before and after PD-1 inhibitor therapy in patients with NSCLC. Moreover, antioxidants are related to the pathogenesis of NSCLC, and serum bilirubin is one of the key factors affecting this process and has a predictive effect on the development of NSCLC in patients. Low levels of serum bilirubin significantly increase the incidence of NSCCL and accelerate the deterioration of patients’ conditions (40).

Finally, based on our experimental data combined with previous research results, we established a predictive model for predicting the early efficacy of combined chemotherapy and PD-1 monoclonal antibody therapy in patients with NSCLC based on peripheral blood indicators. The model in this study underwent internal cross-validation using the leave-one-out method, demonstrating high sensitivity (0.864) but with specificity that requires improvement (0.600). These metrics provide directions for further optimization of the model. For instance, adjustments to model parameters, increasing the number of feature dimensions, or adopting more complex model architectures could be explored to enhance specificity while maintaining or improving sensitivity, thereby achieving better overall classification performance. Future research will build upon these findings to continue exploring possibilities for model improvement, with the aim of achieving more accurate predictive outcomes.

This study has several limitations. First, we detected only peripheral blood cells and immune markers in the early stages after treatment. The response of immune cells may vary during treatment, so the study design should include sample collection from different treatment periods. In addition, animal models and *in vitro* experiments are needed to confirm this conclusion. However, the accuracy of the model predictions also needs validation in clinical cohorts. Despite some limitations, our study suggests that changes in T-cell responses can predict the efficacy of combined chemotherapy and anti-PD-1 immunotherapy.

In summary, our results indicate that combined chemotherapy and anti-PD-1 therapy induces changes in peripheral blood T cells and cytokines in patients who have successfully undergone immunotherapy. A comprehensive predictive model based on peripheral blood indicators can predict the early efficacy of combined chemotherapy and PD-1 inhibitor therapy in patients with NSCLC.

## Data Availability

The raw data supporting the conclusions of this article will be made available by the authors, without undue reservation.
